# Telomere length measures in the Northern Ireland cohort for the longitudinal study of ageing (NICOLA)

**DOI:** 10.1186/s13104-025-07393-y

**Published:** 2025-08-18

**Authors:** Claire Hill, Laura Smyth, Jill Kilner, Katie Quinn, Angie Scott, Charlotte Neville, Frank Kee, Bernadette McGuinness, Amy Jayne McKnight

**Affiliations:** Centre for Public Health, Queen’s University Belfast, Belfast, UK

**Keywords:** Genetic, Epigenetic, Molecular, Cohort, Telomere, RT-PCR, Aging; NICOLA

## Abstract

**Objectives:**

Using blood derived DNA collected from individuals within the Northern Ireland COhort for the Longitudinal study of Ageing (NICOLA), quantitative real-time polymerase chain reaction (RT-PCR) based absolute telomere length measures were derived in triplicate. This data is generated on a subset of participants (*n* = 2,971) within the NICOLA cohort at Wave 1 baseline. NICOLA commenced Wave 3 of data collection in Autumn 2024.

**Data description:**

The NICOLA study recruited approximately 8,500 people from across Northern Ireland, with individuals recruited as representative of the Northern Ireland population. At recruitment, the NICOLA cohort was 45% male and 55% female. Adults were recruited over the age of 50, with approximately 50% of participants aged between 50 and 65, and 10% of participants over the age of 80. Telomere length (TL) values were determined for 2,971individuals (47.7% male) across 33 batches using the Absolute Human Telomere Length Quantification qPCR Assay kit and Roche LightCycler 480 II. Within-batch duplicate measures were established, as were water controls and non-template controls.

## Objective

Using blood derived DNA collected from individuals within the Northern Ireland COhort for the Longitudinal study of Ageing (NICOLA) [1–3], quantitative real-time polymerase chain reaction (RT-PCR) based telomere length measures were derived. This data is an affiliated product of the larger NICOLA cohort, a study lead by the Centre for Public Health at Queen’s University Belfast (https://www.qub.ac.uk/sites/NICOLA/). Characteristics of the NICOLA cohort, as well as descriptions of the DNA extraction protocols used to extract the DNA utilised in this work are described in the 2017 Early Findings Report [4] and the 2021 Health Assessment Report [5].

Briefly, the NICOLA study aims to build a greater understanding of the aging process and its influence on health and well-being. The NICOLA study consists of approximately 8,500 adults over the age of 50 from Northern Ireland. Approximately 50% of participants were aged between 50 and 65 at time of recruitment, with 10% of participants over the age of 80. At baseline recruitment, the NICOLA cohort was 45% male and 55% female. Participants underwent comprehensive assessments, including computer assisted interviews, self-completed questionnaires, physical measurements, and biological sample collection. The study aims to explore many aspects of aging, such as physical and mental health, cognitive function, lifestyle, environmental exposures and social circumstances. Following participants over time, the NICOLA study will gain insights into the factors that influence healthy aging.

One measure currently used to assess ageing is telomere length [6, 7]. Telomere length measures have similarly been determined for additional longitudinal cohorts, such as the Health and Retirement Study (HRS) [8] and The Irish Longitudinal Study on Ageing (TILDA) [9].

The telomere data described here, together with the wealth of health assessment data already collected and generated (such as health measures, disease diagnoses, genetic variation, epigenetic variation and epigenetic clocks) is available for use by researchers globally.

## Data description

In the NICOLA study, Wave 1 was completed for baseline data in 2020/2021 [5], Wave 2 completed in 2023, and Wave 3 commenced in Autumn 2024 with a repeat health assessment and biological sample collection. All Supplementary figures and tables are available at: 10.17605/OSF.IO/ZD37J (NICOLA Data Products – Associated files, Baseline telomere data, Table 1 [10]).

Within a sub-set of the 8,478 NICOLA participants, telomere length measures were determined via quantitative real-time polymerase chain reaction (RT-PCR). DNA used for this assessment were derived from blood samples collected during the Wave 1 health assessment for 3,514 participants [5]. Blood sample handling and DNA extraction were carried out as described previously [5].The telomere data sample subset is 47.7% male, with mean age 64.07. Age and telomere length summary statistics for these 2,971 participants are shown in Supplementary Table 1 [10].

Telomere data was generated by the Queen’s University Belfast Genomics Core Technology Unit. Samples were run using 384 well plates, with each sample run in triplicate on the respective telomere primer and single copy reference (SCR) primer plates. Alongside this, four non-template controls were run per plate, as well as a triplicate of the reference genomic DNA. The following parameters were utilised across runs: Absolute Quantification Number of Fit Points = 3, Telomere Assay Threshold = 0.8 and SCR Assay Threshold = 0.6. For the Noise Band (Standard deviation multiplier), the standard deviation of the background (noise) signals was automatically calculated and the noise band was then set to 5-fold this standard deviation. Samples were run across 33 batches in total. Duplicate samples, blinded to laboratory staff (*n* = 32, one duplicate per batch) were included within 32 batches. Supplementary Table 2 [10] shows the kits and machinery used for data generation. Efficiencies of the qPCR ranged from 68 to 101% (median efficiency: Telomere = 82.6%; SCR = 85.0%).

Supplementary Fig. 1 https://osf.io/ftu8a [10] shows the different average telomere length (ATL) values determined per duplicate sample. For the 32 duplicated samples, the measurement with lowest total telomere length (TTL) error was carried forward. One non-duplicated sample was removed as all Ct values for the telomere primer replicates for this sample were zero. This resulted in 2,971 distinct samples available for analysis. A Shapiro-Wilk normality test revealed that ATL was not normally distributed (p-value = 1.55 × 10^-36^). Supplementary Fig. 2 [10] also indicates the non-normality of the data. Supplementary Fig. 3 [10] shows how average telomere length (ATL) vary across batches.

Water replicates were run every batch. Average telomere length measures determined from these replicates are shown in Supplementary Fig. 4 [10].

The correlation between age and telomere length is shown in Supplementary Fig. 5 [10]. Stratified by gender, the correlations remained similar (Male = -0.22, Female = -0.21). Note that gender in this instance refers to self-reported gender in the self-completed computer assisted personal interview (CAPI).

Please note that the NICOLA study website contains details of all the data that is available through a fully searchable data dictionary: https://www.qub.ac.uk/sites/NICOLA/InformationforResearchers/#requesting-access-to-nicola-data-or-biological-samples-910951-1.

**Table 1:** Overview of data files/data sets

Label	Name of data file/data set	File types (file extension)	Data repository and identifier (DOI or accession number)
Data set 1	Baseline_Telomere_data_NICOLA_n2971_TeloID_only_CH_AJ_290425.csv	.csv	Open Science Framework () [10]
Data file 1	Baseline_Telomere_NICOLA_datanote_supplementary_CH_AJ_290425.pdf	.pdf	Open Science Framework () [10]

## Limitations

- lengths have only been determined for a sub-set of the larger NICOLA cohort, which is based on the availability of high-quality DNA for this analysis from the Wave 1 baseline cohort
- RT-PCR approach only has been used. Other approaches, such as long-read sequencing and Southern blot are available [11], but due to the large quantity of samples analysed (*n* = 2,971), this method was most appropriate with regards to cost effectiveness and throughput. We have previously demonstrated concordance (*r* > 0.8) between telomere length measured by PCR and Southern blot approaches [12]. RT-PCR was also selected due to the small amount of DNA required to obtain telomere length measurements. We have shown very little variability in our blind duplicate samples using this RT-PCR approach
- one batch, a duplicate sample was not available, but we are confident this does not reflect a significant issue as other duplicates demonstrated good concordance

## Data Availability

The data described in this Data note can be freely and openly accessed on the Open Science Framework under DOI 10.17605/OSF.IO/ZD37 (NICOLA Data Products - associated files, Baseline telomere data https://doi.org/10.17605/OSF.IO/ZD37J) [10]. Please see Table 1 for details and links to the data.

## Abbreviations

NICOLA: Northern Ireland COhort for the Longitudinal study of Ageing
TL: Telomere length
HRS: Health and Retirement Study
TILDA: The Irish Longitudinal Study on Ageing
RT: PCR-real-time polymerase chain reaction
TTL: Total telomere length
ATL: Average telomere length
IQR: Interquartile range
